# Outcomes of systemic Janus kinase inhibitors following prior dupilumab use for atopic dermatitis: An evidence-based review

**DOI:** 10.1016/j.jdin.2024.03.023

**Published:** 2024-04-06

**Authors:** Siddhartha Sood, Ahmed Bagit, Martin Heung, Khalad Maliyar, Abrahim Abduelmula, Muskaan Sachdeva, Jorge R. Georgakopoulos, Asfandyar Mufti, Vimal H. Prajapati, Jensen Yeung

**Affiliations:** a Temerty Faculty of Medicine, University of Toronto, Toronto, Ontario, Canada; b Faculty of Health Sciences, McMaster University, Hamilton, Ontario, Canada; c Division of Dermatology, Department of Medicine, University of Toronto, Toronto, Ontario, Canada; d Department of Dermatology, Sunnybrook Health Sciences Centre, Toronto, Ontario, Canada; e Division of Dermatology, Department of Medicine, University of Calgary, Calgary, Alberta, Canada; f Dermatology Research Institute, Calgary, Alberta, Canada; g Skin Health & Wellness Centre, Calgary, Alberta, Canada; h Section of Community Pediatrics, Department of Pediatrics, University of Calgary, Calgary, Alberta, Canada; i Section of Pediatric Rheumatology, Department of Pediatrics, University of Calgary, Calgary, Alberta, Canada; j Probity Medical Research, Waterloo, Ontario, Canada; k Department of Dermatology, Women's College Hospital, Toronto, Ontario, Canada

**Keywords:** abrocitinib, atopic dermatitis, baricitinib, dupilumab, evidence-based, Janus kinase inhibitor, systematic review, upadacitinib

*To the Editor:* Atopic dermatitis (AD) is a common, chronic, highly pruritic, inflammatory condition.^1^ Although several Janus kinase inhibitors (JAKis) have recently been approved for moderate-to-severe AD, limited guidance is available regarding the role of systemic JAKi in patients who have previously utilized dupilumab. We conducted a systematic review to evaluate JAKi outcomes for this specific population.

Following Preferred Reporting Items for Systematic Reviews and Meta-Analyses guidelines, Embase and MEDLINE databases were searched using several keywords (Supplementary Table I, available via Mendeley at https://data.mendeley.com/datasets/mrj57rwdp2/1). Quality of evidence was evaluated using Oxford Centre for Evidence-Based Medicine 2011 Levels of Evidence. After independent screening by 2 reviewers, 20 articles (publication date: 2022-2023) reflecting 518 patients were included (Fig 1; Supplementary Table II, available via Mendeley at https://data.mendeley.com/datasets/mrj57rwdp2/1). The mean age was 36.9 years (range: 12-75 years) with sex reported in 315 patients (161 males [51.1%], 154 females [48.9%]). There were 153 (29.5%) patients reported to be refractory to previous systemic nonbiologic therapy, of which oral corticosteroids were the most commonly used (17%, 26/153). Mean dupilumab treatment duration prior to systemic JAKi use was 160.4 days (502/518) (Supplementary Table II, available via Mendeley at https://data.mendeley.com/datasets/mrj57rwdp2/1).

**Fig 1:**
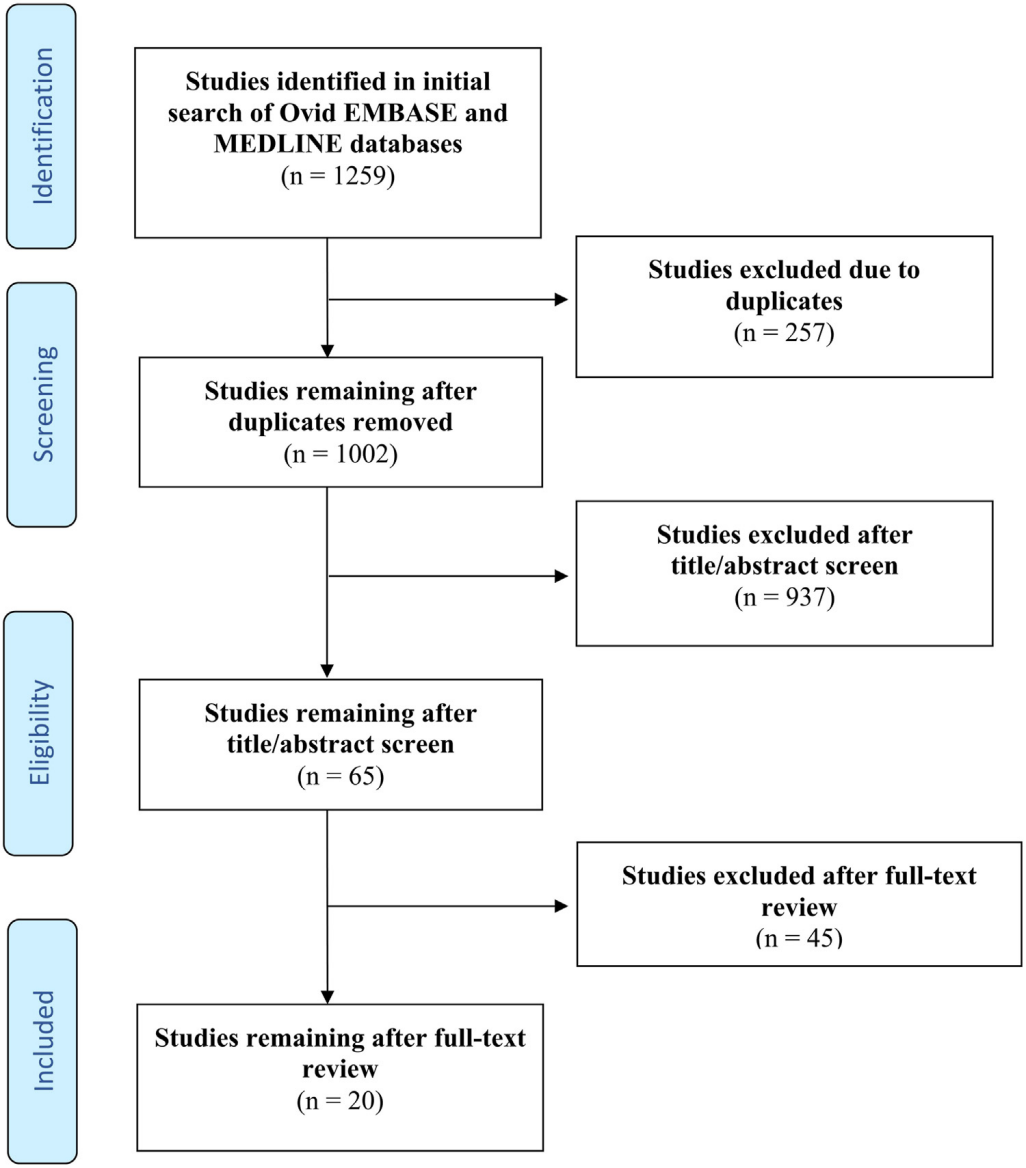
Flow diagram of literature screening using the Preferred Reporting Items for Systematic Reviews and Meta-Analyses guidelines. Figure adapted from http://prisma-statement.org.

There were 518 instances of systemic JAKi treatment following dupilumab use: upadacitinib (58.1%, 301/518), abrocitinib (40.9%, 212/518), and baricitinib (1%, 5/518) (Supplementary Table III, available via Mendeley at https://data.mendeley.com/datasets/mrj57rwdp2/1). Mean systemic JAKi treatment duration was 94 days (507/518). Upadacitinib, abrocitinib, and baricitinib led to improvement in 75.7% (228/301), 58%, (123/212), and 80% (4/5) of cases respectively. No resolution was noted in 1 (20%) case of baricitinib use (Supplementary Table II, available via Mendeley at https://data.mendeley.com/datasets/mrj57rwdp2/1). In patients not achieving Eczema Area and Severity Index (EASI) improvement of 75% (EASI75) with dupilumab, 69.5% (105/151) and 49.6% (67/135) subsequently achieved EASI75 and EASI90 (90% improvement in EASI), respectively with systemic JAKi. Similarly, in patients not achieving EASI90 and EASI100 (100% improvement in EASI) with dupilumab, these outcomes were subsequently achieved by 69.7% (69/99) and 55.6% (65/117), respectively during JAKi treatment (Supplementary Table III, available via Mendeley at https://data.mendeley.com/datasets/mrj57rwdp2/1). Dupilumab-induced adverse events resolved in 97.7% (43/44) of cases, most frequently reflecting head-and-neck dermatitis (61.4%, 27/44) and ocular surface disease (36.4%, 16/44) (Supplementary Table IV, available via Mendeley at https://data.mendeley.com/datasets/mrj57rwdp2/1). In 296 reported instances, 84 (28.4%) adverse events were identified with systemic JAKi use, the most common of which were acne (22.6%, 19/84) and nasopharyngitis (20.2%, 17/84) (Supplementary Table II, available via Mendeley at https://data.mendeley.com/datasets/mrj57rwdp2/1).

Recent consensus-based guidelines by American Academy of Dermatology have recommended dupilumab and systemic JAKi with strong evidence as treatments for moderate-to-severe AD.^2^ Furthermore, head-to-head clinical trials have demonstrated a significantly greater proportion of patients achieving Investigator Global Assessment 0/1 (week 16; upadacitinib), EASI75/EASI90 (week 16; upadacitinib/abrocitinib), and improvements in pruritus Numeric Rating Scale (week 2; upadacitinib/abrocitinib) with certain systemic JAKi in comparison to dupilumab.^1^^,^^3^ Similarly, an open-label extension study noted that 21.2% and 56% of patients not achieving EASI75 and EASI90/EASI100 responses, respectively, with dupilumab subsequently achieved EASI100 with upadacitinib.^4^ These results are in keeping with our review. An additional clinical trial is currently underway evaluating upadacitinib outcomes in dupilumab nonresponders (NCT05394792).^5^

Study limitations include incomplete follow-up data and potential selection bias. Data heterogeneity prevented meta-analysis. Regardless, we highlight evidence on utility of systemic JAKi use following dupilumab for AD. Longer-term studies are warranted.

## Conflicts of interest

Dr Mufti has been a speaker for AbbVie and Janssen. Dr Prajapati has been an advisor, consultant, speaker, and/or investigator for AbbVie, Actelion, Amgen, AnaptysBio, Apogee Therapeutics, Aralez, Arcutis, Arena, Asana, Aspen, Bausch Health, BioScript Solutions, Boehringer Ingelheim, Bristol Myers Squibb, Celgene, Cipher, Concert, CorEvitas, Dermavant, Dermira, Eli Lilly, Galderma, GlaxoSmithKline, Homeocan, Incyte, Janssen, LEO Pharma, Medexus, Nimbus Lakshmi, Novartis, Pediapharm, Pfizer, RAPT Therapeutics, Regeneron, Reistone, Sanofi Genzyme, Sun Pharma, Takeda, Tribute, UCB, and Valeant. Dr Yeung has been an advisor, consultant, speaker, and/or investigator for AbbVie, Allergan, Amgen, Astellas, Boehringer Ingelheim, Celgene, Centocor, Coherus, Dermira, Eli Lilly, Forward, Galderma, GSK, Janssen, LEO Pharma, Medimmune, Merck, Novartis, Pfizer, Regeneron, Roche, Sanofi Genzyme, Sun Pharma, Takeda, UCB, Valeant, and Xenon. Author Sood, Author Bagit, Author Heung, Dr Maliyar, Dr Abduelmula, Dr Sachdeva, and Dr Georgakopoulos have no conflicts of interest to declare.
